# Formulation Strategy for the Delivery of Cyclosporine A: Comparison of Two Polymeric Nanospheres

**DOI:** 10.1038/srep13065

**Published:** 2015-08-13

**Authors:** Ritu Goyal, Lauren Macri, Joachim Kohn

**Affiliations:** 1 New Jersey Center for Biomaterials, Rutgers University, Piscataway, NJ-08854.

## Abstract

A wide range of nanoparticles has been explored for the delivery of highly hydrophobic drugs, but very few publications provide comparative data of the performance of different nanoparticles. To address this need, this publication compares poly(lactic-co-glycolic acid) (PLGA) nanoparticles and nanospheres made from tyrosine-derived tri-block copolymers (termed TyroSpheres) for their respective performance as carriers for cyclosporine A (CSA). Using previously reported data on PLGA, we followed similar experimental protocols to evaluate the *in vitro* characteristics of TyroSpheres. Although there are some similarities between the two particle systems for the delivery of CSA, such as effective encapsulation and epidermal skin penetration, several differences were notable. First, the methods of preparation were different, i.e., self-assembly and emulsion-diffusion-evaporation process for TyroSpheres and PLGA, respectively. Second, TyroSpheres provided 7-day diffusion-controlled release, whereas PLGA nanoparticles provided >21-day erosion-controlled release. Third, the size of TyroSpheres was measured to be ~60–70 nm irrespective of drug loading, whereas the size of PLGA nanoparticles (~100–250 nm) was dependent on drug loading and the method of preparation. Overall, this publication provides a direct comparison between two different types of nanoparticles and illuminates the respective advantages and disadvantages, using CSA as a model for the release of highly hydrophobic drugs.

Nanoparticles have been developed as a promising approach to increase the bioavailability, solubility, circulation time, and resistance to metabolic degradation of lipophilic drugs and deliver these drugs into targeted organs or cells^1^,^2^,^3^. Among the many nanoparticle drug delivery systems (solid lipid and polymeric) reported in the literature^4^,^5^, nanoparticles made of poly(lactic-co-glycolic acid) (PLGA) have been studied extensively. PLGA is one of the most widely used polymeric biomaterials due to its (i) biodegradability and biocompatibility^6^, (ii) track record for Food and Drug Administration (FDA) and European Medicine Agency approval in drug delivery systems^7^, (iii) well described formulations and methods of production, (iv) ability to incorporate hydrophilic and hydrophobic molecules of various sizes^8^, (v) ability to protect drugs from degradation and provide sustained release^9^, and (vi) potential to provide better interaction with biological materials through the modification of its surface properties and targeting to specific organs or cells^10^,^11^. PLGA nanoparticles have been used as drug delivery vehicles for oral and parenteral administration^12^,^13^,^14^,^15^. In 2001, a detailed review of their utility in topical drug delivery applications was published by de Jalon and coworkers^16^. Despite these advantages, there are reports in the literature that PLGA nanoparticles have the potential to induce toxicity from dose dumping^17^, inconsistent release and drug-polymer interactions^18^.

Our laboratory has developed a nanoparticle system that is synthesized from a family of fully-degradable, ABA-type tri-block copolymers made of poly(ethylene glycol), oligomers of desaminotyrosyl-tyrosine esters and suberic acid (Fig. 1A)^19^,^20^. These tyrosine-derived copolymers are amphiphilic and spontaneously self-assemble to form supramolecular nanoparticles with a core-shell architecture in aqueous environments. Preparation via self-assembly is simple, time efficient and the particles exhibit a very low critical aggregation concentration (cac) of 2.6 × 10^−7^ g/mL^19^,^20^. The lipophilic drugs are incorporated into the hydrophobic core, which serves as a drug reservoir, and the hydrophilic shell enables stable dispersion in an aqueous environment^21^. In addition, the hydrophilic shell also offers prolonged circulation time, protection from protein adsorption and subsequent opsonization and clearance from the body^22^. The tyrosine-derived nanoparticles (referred to herein as TyroSpheres) have been shown to be nontoxic *in vitro*^19^ and *in vivo*^23^,^24^, bind and deliver lipophilic drugs^21^, substantially enhance the permeation of lipophilic agents into the epidermis^25^, and have been added to a gel formulation to maximize contact with the skin and prevent the “run-off” effect of non-viscous solutions or suspensions^26^.

Most often, one laboratory reports on multiple variations of the same basic nanoparticle system. For example, Sheihet *et al.* (2007) demonstrated that changes in the tyrosine-derived tri-block copolymer composition affect drug loading capabilities^21^. Knop *et al.* (2013) compared two amphiphilic star-shaped polymers, poly(ε-caprolactone)-block-poly(oligo(ethylene glycol)methacrylate)s and poly(ε-caprolactone)-block-poly(oligo(2-ethyl-2-oxazoline)methacrylate)s and showed that the molar mass of the macromonomer rather than the mere length was the crucial factor to form an efficiently stabilizing hydrophilic shell^27^. Zhao *et al.* (2013) compared the capability of 4-arm and 6-arm mPEG-PLA micelles to deliver a hydrophobic drug, indomethacin, and found that loading was higher in the 4-arm than the 6-arm copolymer^28^. According to our knowledge, there are only few literature reports available that directly compare two or more different nanoparticle formulations for the delivery of a specific drug. One study by Quan *et al.* (2014) showed a head-to-head comparison of four variations of a nanoparticle system for the delivery of dexamethasone in an adjuvant-induced arthritis rat model: liposomes, core-cross-linked micelles, slow releasing polymeric prodrugs, and fast releasing polymeric prodrugs^29^. In a second study, Abdel-Mottaleb *et al.* (2011) presented a direct comparison of two dissimilar nanoparticular systems (polymeric versus lipid-based) and showed that lipid and polymeric nanoparticles were more efficient for transdermal drug delivery and localized drug delivery to the dermis, respectively^30^. In a third study, Daman *et al.* (2014) demonstrated that self-assembled formulations promoted higher *in vitro* cellular uptake and cytotoxicity when compared to micellar formulations of the same polymer^31^.

In our work, we directly compared two different types of polymeric nanoparticles and their respective performance: TyroSpheres and PLGA nanoparticles. A recent report by Jain *et al.* on the topical delivery of CSA using PLGA nanoparticles provides a rich collection of characterization and performance data^32^,^33^. This made it possible for us to conduct a study, comparing PLGA nanoparticles (based on Jain’s published data) with the TyroSphere system. To facilitate this comparison, we adapted and followed as much as possible the procedures described by Jain *et al.*^32^. Specifically, we used the same drug (CSA) and the same target application (enhancement of skin penetration), and conducted similar *in vitro* evaluation studies (particle fabrication, particle size and distribution, drug release kinetics, stability, and skin distribution profile).

## Results and Discussion

The polymer was synthesized using previously published protocols and polymer composition was confirmed using ^1^H-NMR^21^. The data obtained on CSA-loaded TyroSpheres was compared to two reports by Jain *et al.* on the evaluation of PLGA nanoparticles for CSA delivery^32^,^33^.

### Characterization of nanoparticles

#### Nanoparticle preparation methods: Self-assembly vs. emulsion-diffusion-evaporation

The methods used to prepare TyroSpheres (self-assembly) and PLGA nanoparticles (emulsion-diffusion-evaporation) are substantially different. The preparation of CSA-TyroSpheres is a simple, single step method that depends on the self-assembly of a tri-block (ABA) tyrosine-derived copolymer in aqueous solution. In this method, the ABA-tri-block tyrosine-derived copolymer is added drop-wise to an aqueous solution containing CSA, and self-assembles into a nano-particle. No use of stabilizers or sonication/homogenization is required as opposed to other methods of nanoparticle preparation^34^.

The emulsion-diffusion-evaporation method used by Jain *et al.* results in the formation of nano to micron sized particles depending on the speed of shearing used in the process. Although this method results in the formation of spherical particles, which can be tuned in terms of their size and morphology, it requires the use of a stabilizer (such as poly(vinyl alcohol) or surfactants) for the prevention of aggregation or to increase the shelf life of the particles^32^.

Although both methods form spherical particles that can be tailored for size and morphology, the preparation method for TyroSpheres and the nature of the polymer offer several advantages. First, the TyroSpheres are prepared using a simpler, single-step method as compared to the PLGA nanoparticles, which requires a multi-step method and a stabilizer (e.g., poly(vinyl alcohol), surfactants, etc.). Second, the nature of the polymer composition (ABA tri-block) efficiently restricts the encapsulation of the lipophilic drug within the hydrophobic core of the TyroSpheres. This minimizes the amount of surface-bound drug, thus preventing a large bolus of drug from bursting out and leading to more controlled release. Third, the copolymer composition intentionally uses molecules of PEG to flank the oligomers of desaminotyrosyl-tyrosine esters and suberic acid. This chemical composition has been shown to decrease opsonization, which can result in increased drug retention time in the blood and reduced nonspecific distribution^35^,^36^.

#### Drug loading content and loading efficiency

Figure 2 shows the drug loading content and loading efficiency of CSA into TyroSpheres for theoretical loading content ranging from 10 to 60 wt.%.

The loading efficiency was consistent at approximately 50%, irrespective of the theoretical loading content (10–60 wt.%). The 50% loss in drug concentration is attributed to the loss of both drug and polymer during the two washing and filtration steps. Despite this loss, the measured (actual) loading content equals the theoretical loading content (up to 40 wt.%), which suggests that the nanoparticles surviving the preparation process are loaded with the targeted concentration of CSA. In the case of 60 wt.% loading, the saturation limit was reached which caused the drug to precipitate, ultimately resulting in an average actual loading content of 35% with large variability (Table S1, see supplementary data). Based on these data, subsequent characterization was performed using TyroSpheres loaded with 30 wt.% CSA.

In contrast, the actual loading efficiencies of CSA into PLGA nanoparticles varied with theoretical loading concentrations. Jain *et al.* showed that loading efficiency increased to 86% only up to 10 wt.% target CSA loading and then decreased to 62% efficiency for 15 wt.% theoretical CSA loading. Although the range of theoretical loadings was quite limited (5, 10, and 15 wt.%), these data suggest that the actual loading of CSA into PLGA nanoparticles is substantially more limited than that of the TyroSpheres^32^.

#### Characterization of CSA within nanoparticles

The characteristic peak of CSA within the TyroSpheres was evaluated using XRD. Figure 3 shows the XRD patterns of the tri-block copolymer, empty TyroSpheres, 30 wt.% CSA-TyroSpheres, physical mixture of CSA and tri-block copolymer, and pure CSA. Characteristic peaks for the tri-block copolymer were observed in all samples containing polymer: empty TyroSpheres, polymer, physical mixture of CSA and polymer, and CSA-TyroSpheres (Fig. 3). These data confirm that CSA was successfully loaded into the cavity of the TyroSpheres, and was not present on the surface of the particles.

An explanation for the lack of a CSA peak in the CSA-TyroSpheres sample is that CSA is molecularly dispersed within the hydrophobic polymer domains and hence, does not scatter X-rays efficiently^37^. Similar data was observed by Jain *et al.* for the encapsulation of CSA by PLGA nanoparticles^32^.

#### Size, size distribution, and morphology

The hydrodynamic diameter of empty and CSA-TyroSpheres in aqueous solution was analyzed using DLS. Table S1 (see supplementary data) shows that the diameter ranges from 65–75 nm, irrespective of CSA loading up to 40 wt.%. It is important to note that the surface charge of the TyroSpheres is neutral, which confirms the presence of PEG on the outer core^23^.

The diameter of TyroSpheres loaded with 60 wt.% differs because the drug precipitated out showing that no more drug can be encapsulated at that given weight ratio. The size distribution remains narrow based on the PDI values, which were in the range of 0.2–0.3. The diameter of CSA-TyroSpheres-gel was ~2 times larger than CSA-TyroSpheres. This difference is likely due to the swelling of particles in the presence of HPMC gel resulting in an increased hydrodynamic diameter^38^. In a previous study, the morphology and diameter of lyophilized TyroSpheres and TyroSpheres-gel were studied using transmission electron microscopy (TEM) and it was shown that the average diameter was ~40 nm irrespective of CSA loading (0.5–5 wt.%) or the presence of HPMC gel^26^. An explanation for this is that TyroSpheres have a fixed cavity size regardless of the method of preparation and the type of stabilizer used, and as a result, there was no effect of drug loading on the particle size. In comparison, Jain *et al.* reported that the diameter of empty PLGA nanoparticles ranged from 100–250 nm, as measured by DLS, using various methods of preparation^32^. Also, the size of the particles was dependent on the kind of stabilizer used. In contrast, the average size of PLGA nanoparticles slightly increased by 9.5% (158 nm to 173 nm) when CSA loading was increased from 5 to 15 wt.%, using a standard method and stabilizer. This shows that the size of PLGA nanoparticles is highly dependent on the type of method and stabilizer used.

**Figure 1:**
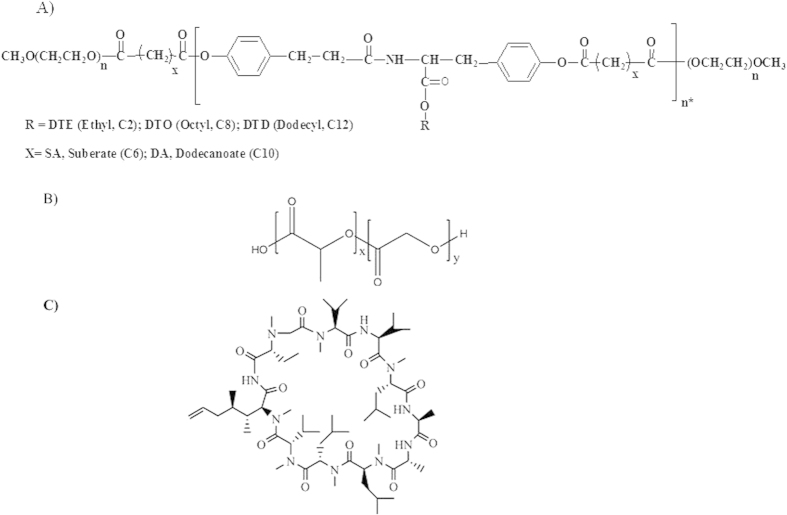
The chemical structures of (**A**) PEG-*b*-oligo(DTR-XA)-*b*-PEG tri-block copolymer, (**B**) Poly-L-lactic-co-glycolic acid (PLGA), and (**C**) Cyclosporine A (CSA).

**Figure 2:**
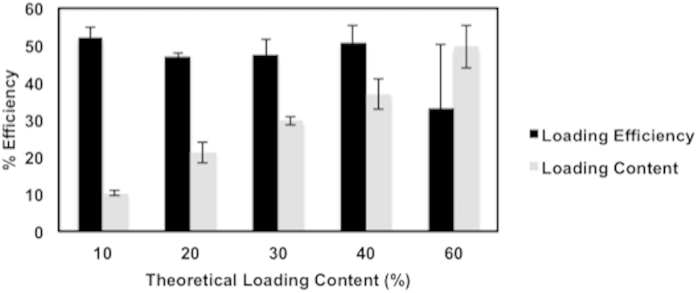
Loading content and loading efficiency of CSA-TyroSpheres with theoretical loading concentrations ranging from 10–60 wt.%.

**Figure 3:**
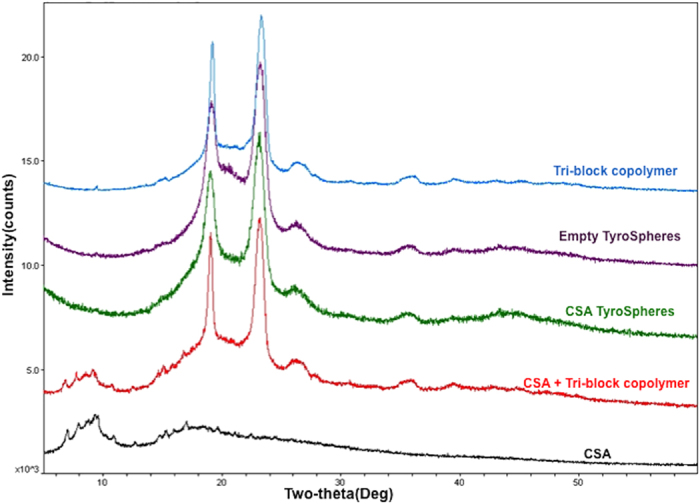
XRD patterns of the tri-block copolymer, empty TyroSpheres, CSA-TyroSpheres, a physical mixture of CSA and the tri-block copolymer, and pure CSA.

Nanoparticles less than 100 nm in size have been shown to enhance the penetration of the cargo into the cells and increase the efficiency of the delivery^39^. In this study, the size of the TyroSpheres was ~65–75 nm irrespective of CSA loading. This indicates that these particles have a fixed cavity size. Additionally, the size of the cavity may be changed by varying the length of the central hydrophobic block in the ABA-tri-block copolymer (Fig. 1A)^40^. On the other hand, the size of PLGA nanoparticles is highly dependent on the type of solvents and stabilizers used during the preparation process. While on one hand this may be an advantage for PLGA-based systems which makes them more tunable, on the other hand, one needs to optimize many parameters such as polymer concentration, type and concentration of stabilizer, speed and time for homogenization, concentration of drug, etc., to reach a targeted particle size^32^. Most data in the literature report a PLGA particle size above 100 nm, however some papers describe methods that can produce particles with diameters lower than 100 nm with or without drug loading^41^,^42^.

### Solubility of CSA

Nanoparticles help in the dispersion of the drug into aqueous solutions by encapsulation and via hydrophobic-hydrophilic interactions^39^. For example, in the case of the self-assembly of the tri-block copolymer, the amphiphilic nature of the polymer allows for encapsulation of the hydrophobic drug inside the hydrophobic core. The hydrophilic outer surface of the polymer (comprising the PEG blocks) ensures interactions with polar solvents (such as water) and allows improved stabilization through hydrophilic interactions. Since the monomers are neutral, the interactions between PEG-water and the drug-hydrophobic core are not electrostatic. TyroSpheres are stable in water predominantly through hydrophobic-hydrophilic interactions. CSA is a hydrophobic drug and is known to have very low solubility (Log P = 4.12) in aqueous solutions. In this study, the solubility of free CSA in solution was compared to CSA encapsulated within TyroSpheres.

Table S2 shows that the solubility of CSA was ~1106 fold higher when encapsulated in TyroSpheres as compared to its free form in PBS. These data suggest that TyroSpheres are considerably more efficient at solubilizing CSA as compared to 1xPBS.

### *In vitro* release of CSA from TyroSpheres

**Figure 4:**
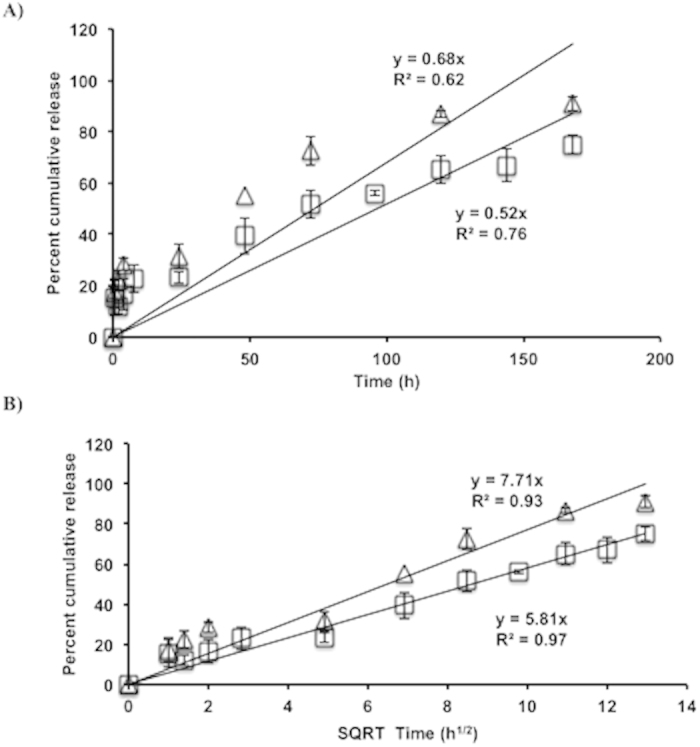
(**A**) *In vitro* release of CSA for 7 days in PBS (pH 7.4, 37 °C) as a function of time from i) 30 wt.% CSA-TyroSpheres (open squares), and ii) 10 wt.% CSA-TyroSpheres (open triangles), (**B**) *In vitro* release of CSA for 7 days in PBS (pH 7.4, 37 °C) as a function of the square root of time (Higuchi model).

Previous reports on the release of hydrophobic moieties from TyroSpheres have shown ~30–40% release within 72 hours (for example, paclitaxel and nile red)^20^,^23^,^25^. The *in vitro* release of CSA from 30 wt.% CSA-TyroSpheres was studied for 7 days in 1xPBS at 37 °C under sink conditions. Figure 4A shows that the release of CSA from CSA-TyroSpheres was sustained for 7 days where 23 ± 1%, 52 ± 1% and 75 ± 10% was released at 1, 3 and 7 days, respectively (R^2^ = 0.76, rate constant, k = 0.52) (Table S3). In addition, the effect of CSA loading (10 vs. 30 wt.%) on the release from TyroSpheres was investigated. Table S3 shows that there was no significant difference (P > 0.05) in the release (R^2^ = 0.62, rate constant, k = 0.68), which indicates that CSA release is not affected by CSA loading in this range. Moreover, the cumulative release was not linear with time (Fig. 4A).

To better elucidate the mechanism of CSA release from the particle systems, data was fit to two rate equations:

Zero order release:


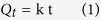


Higuchi model:


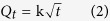


where Q is the amount of drug released, t is time, and k is the rate constant. The release of CSA from TyroSpheres best fit the Higuchi square root model (Equation 2) with R^2^ > 0.9, irrespective of CSA loading. This indicates that the release of CSA from TyroSpheres is diffusion controlled (Fig. 4B). Jain *et al.* demonstrated that the PLGA nanoparticle system provided a longer (>21 vs. 7 days) and slower release of CSA and released only 8%, 15%, and 30% at 1, 3 and 7 days, respectively. In addition, the release profile was reported to have zero order kinetics (Equation 1), suggesting that the release of CSA from these PLGA nanoparticles was predominantly controlled by polymer erosion. However, others have reported that drug release from PLGA nanoparticles takes place by several mechanisms including surface and bulk erosion, disintegration, diffusion, and desorption, where the initial stages of drug release are predominantly controlled by diffusion of the drug, and the later phases by degradation of the polymer matrix^43^.

Overall, the data shows that TyroSpheres provide an initial burst (~23% within 24 h) followed by sustained release (~75% of the payload) of CSA for 7 days. On the other hand, Jain *et al.* showed that PLGA nanoparticles release CSA in a sustained manner for 21 days without an initial burst^32^. Both release profiles could be therapeutically beneficial depending on the targeted clinical indication. For example, a cutaneous wound healing application may benefit from an initial burst to provide immediate relief followed by prolonged release to promote gradual healing^44^. On the other hand, food coatings are used to protect flavors and aromas during processing and storage, but must provide rapid release when the product is consumed^44^.

### Stability of TyroSpheres/CSA-TyroSpheres in solution

**Figure 5:**
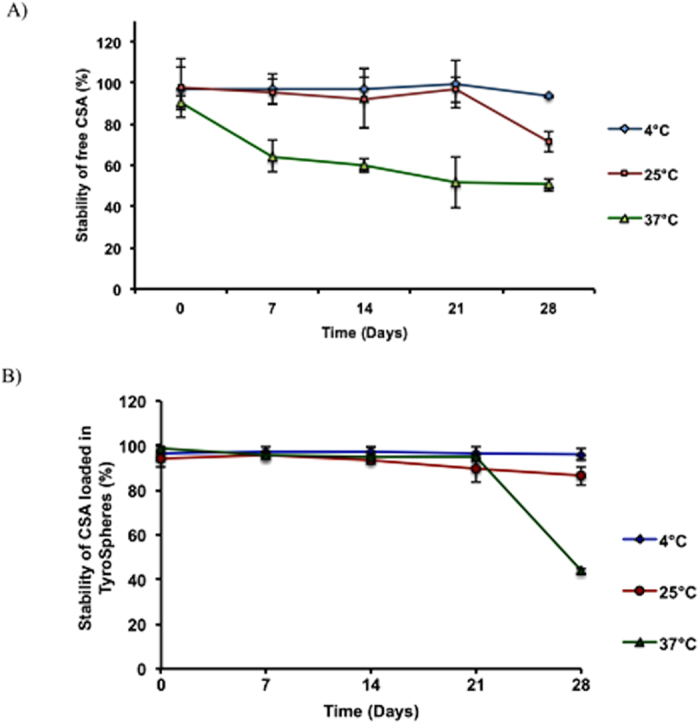
Stability of (**A**) free CSA and (**B**) 30 wt.% CSA-TyroSpheres in PBS for 28 days at 4°, 25° and 37 °C.

No significant difference in particle size was found when unloaded TyroSpheres were suspended in 1xPBS for 6 months at 4 °C: size = 65 ± 5 nm, PDI = 0.18 ± 0.05 at time 0, and size = 68 ± 7 nm, PDI = 0.17 ± 0.08 at 6 months (P > 0.05). Next, the stability of 30 wt.% CSA-TyroSpheres suspended in 1xPBS was assessed for 28 days at 4, 25, and 37 °C and compared to the stability of free CSA (not encapsulated in TyroSpheres). At all storage temperatures, the stability of encapsulated CSA was considerably longer than free CSA (Fig. 5). Specifically, TyroSpheres protected CSA from degradation at 4 °C and 25 °C up to four weeks and at 37 °C up to three weeks. Significant degradation was measured at 37 °C, where 50% of both encapsulated CSA and free CSA degraded at 4 weeks (P < 0.05). In contrast, PLGA nanoparticles are not stable in solution for periods longer than a few days^45^,^46^. Therefore, PLGA-based formulations often require stabilizers when used in solution and are transformed into dry for long-term stability^45^,^46^.

### Freeze-thaw analysis of TyroSpheres

An analysis was done to determine the stability of empty TyroSpheres with and without 10% sucrose (used as a lyoprotectant) suspended in PBS after three freeze-thaw cycles (-80°C, 25°C). Particle size was measured before and after each freeze-thaw cycle and the ratio of the final particle size (Sf) to initial size (Si) was calculated. The Sf/Si ratio was calculated to be close to 1, which suggests minimal change in the particle size as a result of the freeze-thaw cycles (Table 1). Further, the presence of sucrose as a lyoprotectant did not notably change the Sf/Si ratio. Therefore, a stabilizer is not required to provide stability to the liquid formulations.

### Preparation of dry formulations of TyroSpheres

Most nanoparticle formulations are suspensed in aqueous media for application *in vivo*. In addition, many polymeric nanoparticles, including TyroSpheres and PLGA nanoparticles, are susceptible to non-enzymatic hydrolytic degradation that is largely responsible for their instability in aqueous environments. Therefore, the development of a fully re-constitutable, dry formulation of nanoparticles is a suitbale method to increase product shelf-life. A freeze-drying technique was developed to convert aqueous suspensions of TyroSpheres into dry-formulated lyocakes. The optimized freeze-drying process was developed by B. Kilfoyle (PhD Thesis, 2011, New Jersey Center for Biomaterials, Rutgers University) and is summarized in section 2.12 of the materials and methods section of the thesis. This process requires the inclusion of a lyoprotectant (e.g., sugar) to provide stability during the drying process. In this way, the lyoprotectants interact with the nanoparticle surface via hydrogen bonding, displace water molecules during sublimation, prevent nanoparticle destruction, and maintain the nanoparticles’ properties upon rehydration. Various investigators have reported on the use of sugars as lyoprotectants during the freeze-drying process. Layre *et al.* determined that the incorporation of glucose, trehalose, maltose, and saccharose was insufficient for a reduction in agglomeration of polycaprolactone-PEG nanoparticles^47^. Saez *et al.* showed that sucrose, glucose, and trehalose maintained the integrity of PLGA nanoparticles without aggregation during freeze-thaw analysis but after lyophilization, the Sf/Si ratios were above the limit for non-significant changes (1.5)^45^.

**Figure 6:**
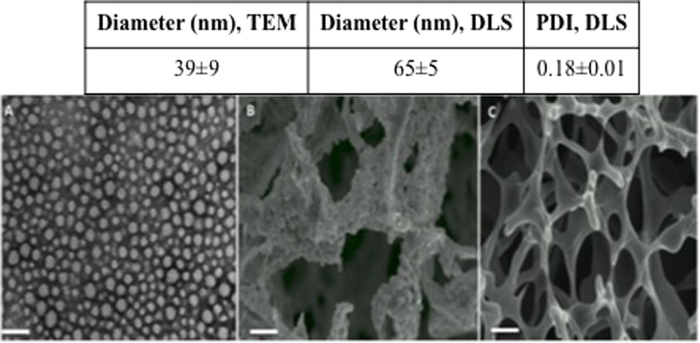
(**A**) A TEM micrograph of TyroSpheres suspended in water. SEM micrographs of lyocakes of TyroSpheres (**B**) without sucrose and (**C**) with 275 mM sucrose as a lyoprotectant. The table included in the figure shows the particle size measured using TEM and DLS. Scale bar = 100 nm for TEM and 10 μm for SEM micrographs.

In this work, we investigated the effects of sucrose concentration as a lyoprotectant for TyroSpheres during freeze-drying (Table S4). Overall, it was observed that TyroSphere particle size (Si) increased with increasing sucrose concentration. Therefore, 275 mM sucrose was determined to be the optimal concentration based on the initial particle size (<100 nm) and the Sf/Si ratio was close to 1 (1.1). Figure 6A shows a TEM micrograph of TyroSpheres suspended in water, where the TyroSpheres in solution are well dispersed and can be individually identified. The table in Fig. 6 contains the particle size measured using TEM micrographs (Image J software, National Institute of Health, USA) and DLS analysis.

Figure 6B,C show SEM micrographs of the TyroSpheres lyocake without and with 275 mM sucrose, respectively. The structure of the lyocake containing TyroSpheres without sucrose shows massive aggregation and a collapsed structure, which is supported by the very high Sf/Si ratio (88). On the other hand, the lyocakes containing TyroSpheres with 275 mM sucrose had a desirable cage-like structure and a Sf/Si ratio of 1.1. It is likely that the sucrose molecules interacted with the nanoparticle surface (via hydrogen bonding) and prevented their collapse^45^.

In a previous study by Saez *et al.*, 20% glucose (w/v) or 20% sucrose (w/v) were found to be the most suitable lyoprotectants for stabilization of cyclosporine A-loaded PLGA nanoparticles^45^. On the other hand, Jain *et al.* used poly(vinyl alcohol) as a stabilizer within the liquid formulation and mannitol or dextrose (2.5 wt.%) as a lyoprotectant for making dry formulations which resulted in a sf/si ratio of ~1.1^32^. Because various research groups have reported different findings with different sugars^32^, a more detailed study is required in this area to gain more insight into the use of lyoprotectants and their mechanism of action.

### Penetration studies of CSA into cadaver skin via TyroSpheres-gel

**Figure 7:**
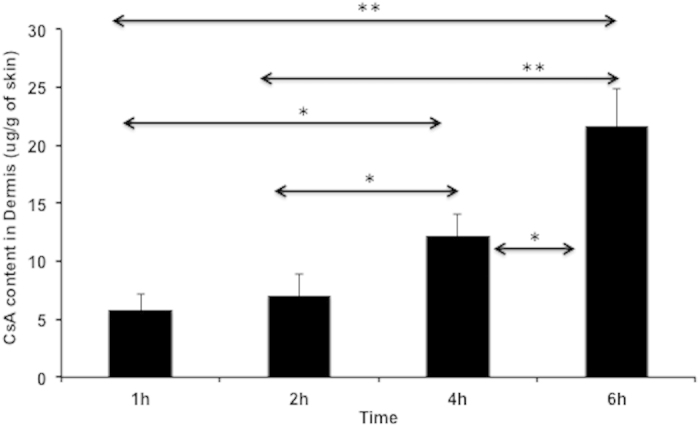
Penetration of CSA eluted from 30 wt.% CSA-TyroSpheres gel through the epidermis and into the dermis of cadaver skin as a function of time. *p < 0.01, p < 0.001.

Skin distribution studies were used to quantify the penetration of CSA into the dermal layer of cadaver skin (Fig. 7). After 6 h of treatment with 30 wt.% CSA-TyroSpheres-gel, ~22 μg of CSA penetrated through the epidermis and into the dermis. CSA-TyroSpheres-gel has an increased viscosity that allows for easy topical application of the formulation without run-off^26^. No CSA was detected in the receptor compartment, suggesting that CSA did not penetrate through the dermis and into the systemic compartment. These results are similar to a previous report by Black *et al.* where 32 μg of CSA was detected in the dermis with negligible systemic exposure when topical CSA was applied mixed with an oil-based vehicle^48^. It is important to note that Black *et al.* used a CSA dose of 25 mg/mL which is ~3 fold higher compared to the TyroSpheres-gel dose of ~8 mg/mL. Also Black *et al.* tested the oil-based formulation in a live mouse model, whereas TyroSpheres were applied to human cadaver skin. These data suggest that the CSA-TyroSpheres formulation holds promise as a topical drug delivery system for dermatological diseases where no systemic exposure is required. Our results are also comparable to Jain *et al.*, who showed that ~16 μg of the total drug content was found in the epidermal and dermal portions of the skin after 24 h^33^. All the values were found to be statistically significant (*p < 0.05, **p < 0.005) except between the 1 h and 2 h time point (values indicated on Fig. 7). These results show that both the formulations may find applications in the clinical setup for use in therapies requiring topical immunosuppression.

The performance of two particle systems – PLGA nanoparticles (formed via emulsion-diffusion-evaporation technique) and TyroSpheres (formed via self-assembly) was compared as topical delivery vehicles for cyclosporine A (CSA). CSA was used as a model drug because of its potential clinical use as a topical immunosuppressive agent in the treatment of various dermatological, immunological diseases^49^,^50^. Our data obtained for drug loading efficiency, release kinetics, effect of stabilizers and skin distribution profiles using TyroSpheres was directly compared to two recent reports by Jain *et al.*^32^,^33^, who reported on the use of PLGA nanoparticles as a topical drug delivery vehicle for CSA. It was observed that while TyroSpheres had the following properties (i) simple and single step self-assembly technique and no use of a stabilizer within the formulation, (ii) flexibility of loading varying amounts of drug into TyroSpheres, (iii) stability in both liquid and dry formulations, and (iv) less than 100 nm sized particles; PLGA was found flexible in terms of (i) choice of solvents and wide range of particle size and (ii) longer and sustained linear release profile. Also, both particle systems are able to deliver hydrophobic drugs to the upper layers of skin without any systemic exposure.

## Materials and Methods

### Materials

CSA was purchased from LC Laboratories (USA). Acetonitrile (ACN) [high performance liquid chromatography (HPLC) grade] and water (HPLC grade) were purchased from Fisher Scientific (Pittsburgh, PA). Dulbecco’s phosphate buffered saline (PBS, pH 7.4) was purchased from Aldrich Chemical Co. (Milwaukee, WI). N,N’-dimethylformamide (DMF) was obtained from Merck (EM Science, Darmstadt, Germany). Hydroxypropylmethylcellulose (HPMC) with 22% methoxyl and 8.1% hydroxypropyl content was purchased from Sigma (St. Louis, MO). All the experiments were carried out in accordance with the approved guidelines from New Jersey Center for Biomaterials.

### Preparation of TyroSpheres loaded with CSA

ABA tri-block copolymer, PEG5K-b-oligo(desaminotyrosyl-tyrosine octyl ester suberate)-b-PEG5K (Fig. 1A), was synthesized, characterized, and prepared using previously established protocols^21^. Briefly, TyroSpheres loaded with and without CSA (referred to as CSA-TyroSpheres or empty, respectively) were prepared as follows. 20% (w/v) polymer (e.g., 60 mg/0.3 mL) and 10, 20, 30, 40 and 60 wt.% CSA (e.g., 20 mg/mL, equivalent to 10 wt.% initial loading) were dissolved in DMF, mixed in equal amounts (0.3 mL each) and added drop-wise to 1xPBS (14.4 mL) under constant stirring. The resulting suspension was passed through 0.22 μm PVDF filters (Millipore) and ultracentrifuged for 3 h at 18 °C and 65,000 rotations per minute (rpm) (290 000 × g ) (Beckman L8-70M ultracentrifuge, Beckman Coulter, Fullerton, CA). The supernatant was aspirated and the CSA-TyroSpheres were rinsed twice with 1 mL 1xPBS. Then, the CSA-TyroSpheres were re-suspended in 1 mL 1xPBS, ultracentrifuged under the same conditions, and re-suspended in 1 mL 1xPBS (pH 7.4) overnight at 25 ± 2 °C.

### Characterization of CSA loaded TyroSpheres

#### Nanoparticle hydrodynamic diameter

The hydrodynamic diameters of the TyroSpheres with and without CSA were obtained by dynamic light scattering (DelsaNano S, Beckman Coulter Inc., NJ) as previously described^25^.

#### Determination of CSA concentration using HPLC

A 100 μL aliquot of CSA-TyroSpheres was lyophillzed and extracted into 2 mL of acetonitrile and filtered using 0.44 μm filters into the HPLC vials. The samples were analyzed on HPLC using the following parameters and a standard curve to determine the concentration of CSA in TyroSpheres. A Waters 2695 HPLC and Empower Pro software were used to quantify CSA in solution. Optimized chromatographic conditions included: column - Waters-C18, 2.1 mm × 50 mm, 5 μm particle size; column temperature −60 °C; injection volume −10 μL; detection wavelength −210 nm; mobile phase—A = HPLC water : B = acetonitrile (isocratic A:B (37.5:62.5)); flow rate −0.5 mL/min; run time −8 minutes; sample diluent - acetonitrile. The limit of detection was determined to be 1 μg. Concentrations of standards of CSA in acetonitrile ranged from 500–1.95 μg/mL.

### Drug loading content and loading efficiency

Loading efficiency of CSA into TyroSpheres was determined by taking 0.1 mL aliquots of CSA-TyroSpheres (with various initial loadings), empty TyroSpheres and PBS and adding them to pre-weighed vials. The samples were frozen on dry ice and lyophilized (Labconco, MO, USA) for 24 h. The samples were re-weighed to determine the mass of salts, mass of empty TyroSpheres and mass of CSA-TyroSpheres. The concentration of CSA loaded in TyroSpheres was determined by adding 2 mL of acetonitrile to the lyophilized CSA-TyroSphere samples followed by vigorous vortexing for 1 h. The samples were then filtered using 0.45 μm filter (Millipore, USA) into HPLC vials. CSA was then quantified using HPLC and the standard curve. Drug loading content and loading efficiency were calculated using the following equations:


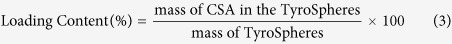



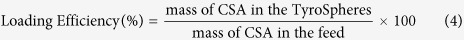


### Solubility of CSA in PBS and TyroSpheres

Excess CSA was added to 1xPBS and stirred on a plate for 72 h at 37 °C in a water bath. The samples were filtered through 0.44 μm filters (PTFE, Puradisc Syringe Filters, Fisher Scientific) to remove non-solubilized drug, lyophilized, and re-dissolved in acetonitrile. The concentration of CSA dissolved in 1xPBS and TyroSpheres was determined as described above.

### Release of CSA from TyroSpheres

The release of CSA from TyroSpheres was investigated using 0.5 mL dialysis cassettes with a 10,000 molecular weight cut off cellulose dialysis membrane (Slide-A-Lyzer, Fischer). The CSA-TyroSpheres were diluted with PBS to a CSA concentration of 400 μg/mL and 0.5 mL was added to the dialysis cassettes. The loaded cassettes were submerged in 200 mL of PBS (pH 7.4) and incubated in a shaking (60 rpm) water bath at 37 °C. At pre-determined time points for up to 7 days, the contents of the dialysis cassettes were collected, the compartment of the dialysis cassettes was rinsed three times with fresh PBS and added to the collected samples. At 24 h intervals, the receptor media was replaced with 200 mL fresh PBS. The concentration of CSA extracted from the donor compartment was determined as described above.

### Preparation of 30 wt.% CSA-TyroSpheres-1% (w/v) HPMC gel formulation

#### Preparation of 1.5% (w/v) HPMC gel

150 mg of HPMC was suspended in 10 mL of 1xPBS and stirred at 400 rpm (slow enough to avoid the formation of bubbles) for 48 h at 25 ± 2 °C.

#### Preparation of 30 wt.% CSA-TyroSpheres-1% (w/v) HPMC gel

5 mL CSA-TyroSpheres were syringe filtered (0.22 μm, Millipore, USA) and mixed with 1.5% (w/v) HPMC gel (10 mL) to create a suspension of 30 wt.% CSA-TyroSpheres in 1% (w/v) HPMC gel (referred herein as CSA-TyroSpheres-gel). The suspension was stirred for 18 h at room temperature and stored at 4 °C until use.

### Penetration studies of CSA-TyroSpheres-gel into cadaver skin

Human cadaver skin (skin bank number: LZ050911, split thickness, male) was cut into twelve 1 inch × 1 inch pieces. Each piece was soaked in PBS at 25 ± 2 °C for 45 min. The skin was then placed on a Franz cell with the epidermis facing the donor compartment. 5 mL 1xPBS was added to the receptor compartment with no air bubbles. 0.5 mL of CSA-TyroSpheres-gel was added to the donor compartment and placed on a 37 °C heating block for 1, 2, 4 and 6 h (n = 3). At each time point, the CSA-TyroSpheres-gel remaining on the skin was removed, the skin was washed several times with 1xPBS, and the dermis was manually separated from the epidermis using a surgical blade and a pair of scissors. Both the donor compartment and receptor compartment were collected and lyophilized for mass balance. The dermis and epidermis from each time point were soaked in 5 mL methanol for 48 h at 37 °C and homogenized (Polytron, Fisher Scientific, PA, USA) thereafter at 4 °C. The solution was then passed through a 0.45 μm filter and analyzed via liquid chromatography-mass spectrometry (LC-MS) at the Center for Integrative Proteomics Research at Rutgers University and a standard curve for CSA content.

### Stability of CSA-TyroSpheres in solution

One mL samples of 30 wt.% CSA-TyroSpheres (30 μg/mL) in PBS (pH 7.4) were stored at three different temperatures (4, 25, and 37 °C) for 28 days. At 0 h, 7 d, 14 d, 21 d, and 28 d, the samples were collected, and freeze-dried. 2 mL of acetonitrile was then added to extract the CSA from the TyroSpheres. Samples were shaken on a vortex for 2 h and the concentration of CSA retained by the TyroSpheres as a function of time and storage temperature was determined as described above. Free CSA was dissolved in 100 μLof DMF and then diluted with 900 μL of 1xPBS to make a 30 μg/mL solution. The stability studies were conducted under identical conditions as the CSA-TyroSpheres.

### Freeze-Thaw Analysis

TyroSpheres were prepared as above and mixed with an equal volume of cryo-protectant solution (550 mM sucrose in 1xPBS). Samples (1 mL) were analyzed using DLS to determine the particle size before freezing, placed in scintillation vials, and stored overnight at −80 °C. Samples were then thawed and allowed to equilibrate to 25 ± 2 °C before performing post-thawing particle size analysis. The procedure was repeated twice. The Sf/Si ratio was calculated by dividing the average post-thaw particle size (Sf) by the average pre-freeze particle size (Si).

### Preparation of Dry Formulation

TyroSpheres were prepared as above and mixed with an equal volume of cryoprotectant solution (550 mM sucrose in 1xPBS). Samples were placed in scintillation vials and covered with KimWipes. A dry formulation was prepared using A MillRock Technology LD85 lyophilizer instrument with Opti-Dry Software with the following method. Step 1—. Freezing: Increase from room temperature to −45 °C as fast as possible, maintain −45 °C for 1 h, decrease temperature to −20 °C as fast as possible. Step 2—Annealing: Maintain at −20 °C for 6 h, decrease to −45 °C as fast as possible, and then remain at -45 °C for 0.5 h. Step 3 - Primary drying: Apply pressure 50 mTorr at −45 °C for 18 h, followed by an increase in temperature to −30 °C for 18 h. Step 4—Secondary drying: Maintain −30 °C for 18 h at 50 mTorr.

Following freeze-drying, the samples were removed from the lyophilizer, immediately sealed under nitrogen (to prevent moisture ingress), and placed under desiccation untill use. Lyophilized samples were analyzed for drug content as described above and samples reconstituted with deionized (DI) water were analyzed for particle size.

### Characterization of Dry Formulation

#### Scanning Electron Microscopy (SEM)

Samples were dispersed lightly on tape mounted to aluminum stubs, sputter-coated with gold-palladium (Balzers SCD 004) and imaged with a Scanning Electron Microscope (AMRAY 1830 I). The microstructure of the cake was grossly analyzed for any appearance of collapse.

#### X-ray diffraction (XRD) Analysis

The XRD patterns of CSA, tri-block copolymer, empty TyroSpheres, CSA-TyroSpheres, and a physical mixture of CSA and tri-block copolymer were obtained using an X-ray diffractometer (Phillips XPert powder diffractometer with sample changer).

### Statistical analysis

Statistical analysis, wherever needed, was carried out by Student’s t-test after ascertaining homogeneity of variance and normality of data. A value of p < 0.05 was considered statistically significant.

## Additional Information

**How to cite this article**: Goyal, R. *et al.* Formulation Strategy for the Delivery of Cyclosporine A: Comparison of Two Polymeric Nanospheres. *Sci. Rep.* **5**, 13065; doi: 10.1038/srep13065 (2015).

## Supplementary Material

Supplementary Information

## Figures and Tables

**Table 1:** The effect of freeze-thaw cycles on the particle size of TyroSpheres with and without the presence of sucrose as a lyoprotectant (Mean ± SD is reported, n = 3).

**Sample (n = 3)**	**Before freezing**		**After thawing**		**Sf/Si**
	**Particle size (nm)**	**PDI**	**Particle size (nm)**	**PDI**	
TyroSpheres alone	74 ± 5	0.18 ± 0.01	84 ± 6	0.19 ± 0.01	1.1
TyroSpheres + sucrose	93 ± 3	0.18 ± 0.01	100 ± 4	0.21 ± 0.02	1.1
