# Supplementary material for: Formulation Strategy for the Delivery of Cyclosporine A: Comparison of Two Polymeric Nanospheres
Source: Sci Rep. 2015 Aug 13;5:13065. doi: 10.1038/srep13065 (PMC4535033; doi:10.1038/srep13065)
Supplement: Supplementary Information [file srep13065-s1.pdf]

Supplementary Information

Formulation Strategy for the Delivery of Cyclosporine A: Comparison of Two Polymeric Nanospheres

Ritu Goyal[1], Lauren Macri[1], Joachim Kohn[1]*

[1]New Jersey Center for Biomaterials, Rutgers University, Piscataway, NJ-08854

***Corresponding Author**

Joachim Kohn, Ph.D. FBSE

Email: kohn@dls.rutgers.edu

Ph: +1 7324077510 ext. 53888

Table S1: Particle size of CSA-TyroSpheres using dynamic light scattering

Drug initial Loading wt.% of polymer	Particle Size (nm)±S.D.	PDI±S.D.
0 (Empty TyroSpheres)	65±5	0.18±0.01
10	66±4	0.22±0.03
20	73±4	0.17±0.02
30	71±5	0.27±0.03
40	74±3	0.20±0.01
60 (limit of saturation)	86±5	0.21±0.06

Table S2: Solubility of CSA in PBS and TyroSpheres. Mean ± SD is shown (N = 3)

Solution	Solubility of CSA (µg/mL)±S.D.
1xPBS	8±1
30 wt.% CSA-TyroSpheres suspended in 1xPBS	8743±152

Table S3: Corresponding R^2 and K values of the release curves.

Sample	R^2	K
10wt.% CSA-TyroSpheres	0.62	0.68
30wt.% CSA-TyroSpheres	0.76	0.52
Higuchi model fit -10wt.% CSA-TyroSpheres	0.93	7.71
Higuchi model fit -30wt.% CSA-TyroSpheres	0.96	5.80

**Table S4:** Optimization of sucrose concentration in dry formulation					
Sucrose Concentration (mM)	Diameter of 30 wt.% CSA-TyroSpheres (nm)±S.D.		PDI±S.D. (Before freeze drying)	PDI±S.D (After freeze drying)	Sf/Si
	Before Freeze Drying (Si)	After Freeze Drying (Sf)			
0	76±12	6642±297	0.23±0.02	0.79±0.10	88
100	81±5	174±25	0.19±0.02	0.24±0.04	2
200	87±3	116±12	0.17±0.02	0.26±0.04	1
275	92±4	103±6	0.20±0.03	0.24±0.05	1
300	96±4	110±5	0.18±0.04	0.22±0.04	1
350	104±6	111±5	0.19±0.08	0.25±0.04	1
400	105±6	109±6	0.23±0.02	0.24±0.05	1
